# Change in the Gut Microbiota of Lactating Sows and Their Piglets by Inclusion of Dietary Spray-Dried Plasma in Sow Diets

**DOI:** 10.4014/jmb.2311.11001

**Published:** 2023-12-15

**Authors:** Jeong Jae Lee, Hyunjin Kyoung, Jin Ho Cho, Kyeong Il Park, Yonghee Kim, Jinmu Ahn, Jeehwan Choe, Younghoon Kim, Hyeun Bum Kim, Minho Song

**Affiliations:** 1 Institute of Agricultural Science and Technology, Kyungpook National University, Daegu 41566, Republic of Korea; 2 Division of Animal and Dairy Science, Chungnam National University, Daejeon 34134, Republic of Korea; 3 Division of Food and Animal Science, Chungbuk National University, Cheongju 28644, Republic of Korea; 4 Korea National of Agriculture and Fisheries, Jeonju 54874, Republic of Korea; 5 Department of Agricultural Biotechnology and Research Institute of Agriculture and Life Sciences, Seoul National University, Seoul 08826, Republic of Korea; 6 Department of Animal Resources Science, Dankook University, Cheonan 31116, Republic of Korea

**Keywords:** Dietary spray-dried plasma, fecal microbiota, lactating sows, nursing piglets

## Abstract

This study aimed to investigate the effects of dietary spray-dried plasma (SDP) on the gut microbiota of lactating sows and their piglets. A total of 12 sows were randomly assigned to one of two dietary treatment groups in a completely randomized design. The treatments were a sow diet based on corn and soybean meal (CON), and a CON diet with an added 1% SDP. The sows were fed the dietary treatments from d 30 before farrowing to weaning (d 28). The fecal samples of three sows from each treatment and two of their randomly selected piglets were collected to verify their fecal microbiota. There were no differences in the alpha diversity and distinct clustering of the microbial communities in the sows and their piglets when SDP was added to the sow diets from late gestation to weaning. The fecal microbiota of the lactating sows and their piglets showed a higher relative abundance of the phylum Bacteroidota and genus *Lactobacillus* and *Ruminococcus* and showed a lower relative abundance of the phylum Bacillota and genus *Bacteroides*, *Escherichia/Shigella*, and *Clostridium* in the sows fed the SDP diet than those fed the CON diet. Overall, these results show that the addition of SDP to the sow diet during lactation altered the gut environment with positive microbial composition changes. These results were similar in the nursing piglets, suggesting that the control of the sow diets during lactation may contribute to the intestinal health and growth in piglets after weaning.

## Introduction

Sow feeding management during farrowing and lactation is an important nutritional strategy for pig production [1]. The nutritional status during gestation affects the sow placental quality and fetal development [2-4]. Previous studies have reported that the sow gestation diet supplemented with amino acids such as arginine and glutamine increased the placental blood supply and total litter weight at birth [3, 5]. Moreover, the farrowing and lactation period are physiologically important times because the hormones and metabolites related to milk production are modified by the nutritional changes in sow [6]. Therefore, the nutritional management of sows during lactation is a major factor in determining the quality and yield of breast milk, which modifies the intestinal environment of suckling piglets [7].

The gastrointestinal tract (GIT) of neonatal piglets is underdeveloped at birth and the neonatal gut microbiota is maternally independent. The diarrhea of piglets after weaning is increased by GIT disorders and pathogenic microbial infections, which causes economic loss in the swine industry. The gut microbiota of neonatal piglets is established by the environment with their sow and the various bacteria and prebiotic compounds in the breast milk impact the intestinal development [1, 8, 9]. In suckling piglets, *Lactobacilli*, which use oligosaccharides in the milk as an energy source, is established in the intestine and remains a predominant microbiota [9, 10] . The growth of *Lactobacilli* protects the neonatal piglets from intestinal disease such as diarrhea by inhibiting the growth of pathogenic bacteria [11, 12]. Thus, the gut microbiota composition of neonatal piglets is correlated with the breast milk composition, which is determined by the nutrients in the sow lactation diets.

Dietary spray-dried plasma (SDP) is a by-product of livestock produced after slaughtering porcine, bovine, and chicken, and is considered a feed additive because it contains growth factors such as fibrinogen, albumin, glycoprotein, and immunoglobulin [13].

Moreover, SDP is currently being considered as an alternative to antibiotics in livestock feed because of its antimicrobial effect. Several studies have reported that weaning diets with the addition of SDP increased the number of *Lactobacilli* and reduced the *Escherichia coli* population in the ileal and cecal digesta and blocked the adhesion of pathogenic bacteria to the intestinal epithelium [14-16]. Thus, we hypothesized that the feeding a diet with added SDP to sows during late farrowing and lactation would directly correlate with changes in the gut microbiota of the sows and the establishment of the gut microbiota in their piglets. Therefore, the objective of this study was to investigate the effects of lactation diets with an added 1% SDP on the gut microbiota of lactating sows and their litters.

## Materials and Methods

### Animals, Diets, and Study Design

A total of 12 lactating sows (Yorkshire × Landrace) with an average body weight (BW) of 227.78 ± 2.16 kg and average parity of 2.0 were used in this experiment. The sows were randomly allotted to one of two dietary treatments at d 84 of gestation based on a completely randomized design. On d 109 of gestation, the sows were moved from gestation crates to individual farrowing crates with free access to water. The dietary treatments were a typical diet based on corn and soybean meal as the control diet (CON) and the CON diet with an added 1% SDP. All dietary treatments were formulated to meet or exceed the national research council (NRC) requirements of gestating and lactating sows [17] (Table 1). Sows were fed 3.0 kg of the dietary treatments from d 84 of gestation until farrowing and lactation before weaning with free access to the diet and water.

### Sample Collection and Preparation for Microbial Analysis

Three sow fecal samples per group were collected using rectal palpation at d 109 of gestation (before farrowing, -d 5), d 7, and d 28 (at weaning) of lactation. The feces of their litters were collected on d 7 and d 28 (at weaning) of lactation. The litter fecal samples of each sow were collected from two randomly selected piglets (one male and one female) by rectal palpation. The fecal samples were stored at -20°C until analysis. The DNA in each fecal sample was extracted using the Powerfood Microbial DNA Isolation kit (Mo Bio Laboratories, Inc., USA) according to the manufacturer’s instructions. The concentration and quality of the isolated DNA were assessed using NanoDrop ND-1000 spectrophotometer (NanoDrop Technologies, USA) and stored at -20°C for analysis.

### Fecal Microbiota Analysis by 16S rRNA Gene Sequencing

Each isolated genomic DNA sample was adjusted to a concentration of 1 ng/μl, and the targeting V3-V4 region of the 16s rRNA microbial genes were ampliﬁed by the polymerase chain reaction (PCR) step using specific primers as listed previously [18]. Sequenced primers were designed based on 16S rRNA metagenomic sequencing library [19]. By PCR, Bakt 341F (5-CCTACGGGNGGCWGCAG-3) and Bakt 805R (5-GGACTACHVGGGTWTCTAAT-3) with barcodes were ampliﬁed [19]. The PCR product puriﬁcation was performed by Wizard SV Gel and PCR Clean Up System (Promega, USA). The puriﬁed PCR product was conﬁrmed by 1% agarose gel electrophoresis. The amplicon were sequenced by Illumina MiSeq platform in a commercial company (Macrogen Inc., Republic of Korea). Raw sequence data were processed by the Mothur software, and low-quality sequences were eliminated [20]. Sequencing errors and chimeras were eliminated using UCHIME during Mothur processing [18]. The remaining high quality sequences were categorized into operational taxonomic units (OTUs) clustering according to an identity cutoff of 97% [21]. Bacterial taxonomic composition and community for each sample were performed using UCLUST [22] and Quantitative Insights into Microbial Ecology (QIIME) [23] based on ribosomal database project classiﬁer for 16S rRNA. Alpha diversity was calculated using OTUs, Chao 1, Shannon, and Simpson indices. The principal coordinate analysis (PCoA) of beta diversity was performed by UniFrac distance metrics in QIIME to relate the bacterial community between dietary treatments. The taxonomic composition of each sample at phylum, family, and genus levels was shown as a percentage based on relative abundance.

### Statistical Analysis

Alpha diversity data were expressed as mean ± standard deviation. Microbiota data were analyzed by Student’s *t*-test using GraphPad Prism 9 (USA) with P < 0.05 considered as statistical significance.

## Results

**Fig. 1:**
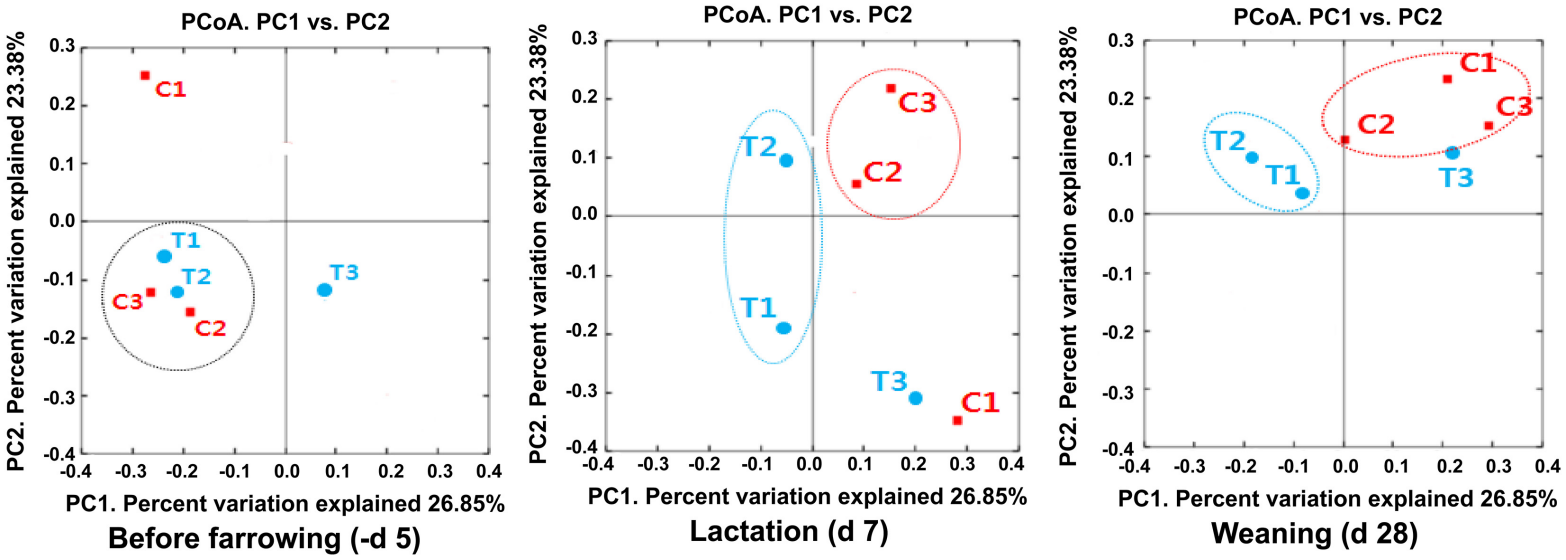
Principal coordinates analysis (PCoA) plot based on the weighted UniFrac distance metrics among the sow fecal samples. *n* = 3; CON, control diet (red); SDP, CON added with 1% dietary SDP (blue).

**Fig. 2:**
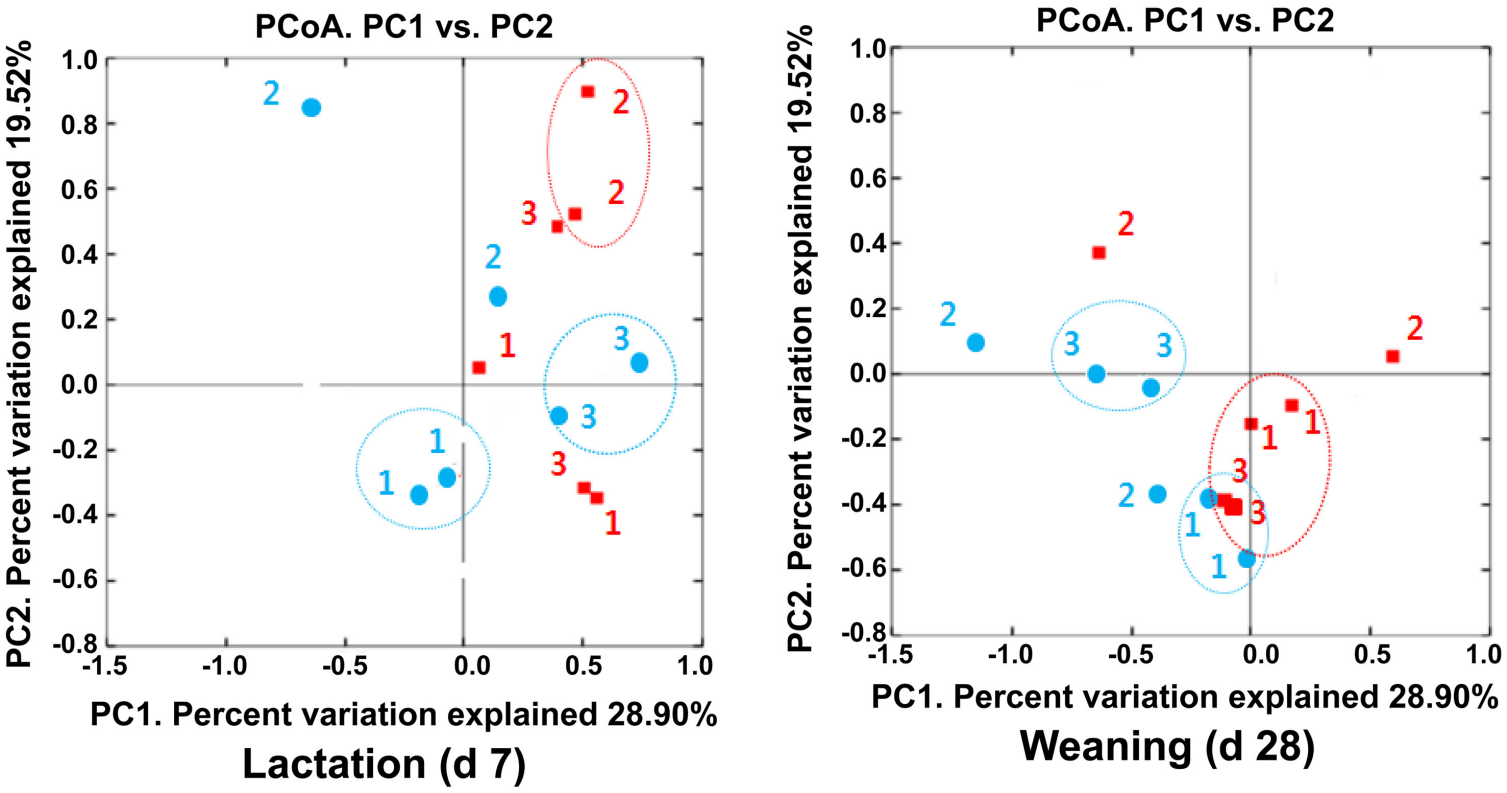
PCoA plot based on the weighted UniFrac distance metrics among the piglet fecal samples. *n* = 3; CON, control diet (red); SDP, CON added with 1% dietary SDP (blue).

Microbial Diversity of the Gut Microbiota of Lactating Sows and Their Piglets Using 16S rRNA Gene Sequencing Fecal samples from the sows and their piglets were analyzed for alpha diversity using 16S rRNA gene sequencing. The six fecal samples from the sows in each dietary treatment group were collected on d 109 of gestation (before farrowing, -d 5), d 7, and d 28 (at weaning) of lactation (Table 2). The total of bacterial sequencing reads were 7,475.67 ± 3,317.83 before farrowing (-d 5), 4,968.33 ± 1,758.36 at d 7 of lactation, and 3,601.00 ± 528.37 at weaning (d 28) with no difference between the dietary treatment groups. Moreover, the differences in the alpha diversity analysis such as the OTUs, Chao 1, Shannon, and Simpson index observed between the CON and SDP dietary treatment groups at each period were not statistically significant. The 12 fecal samples of the piglets in each sow group were collected on d 7 of lactation and d 28 at weaning (Table 3). The total bacterial sequencing reads were 4,389.50 ± 1,157.98 at d 7 of lactation and 4,290.67 ± 2,392.39 at weaning (d 28), but the piglets in the CON group had higher (2613.67 ± 838.39 vs. 1775.83 ± 319.59, *p* < 0.05) average sequencing reads on d 7 of lactation than the piglets in the SDP group. However, there were no differences in OTUs, Chao1, Shannon, and Simpson index observed between the CON and SDP dietary treatment groups. Fecal microbial communities of the sows based on PCoA plots of the weighted UniFrac distances showed extensive clustering between the CON and SDP groups on d 7 of lactation (Fig. 1). However, there were no differences in the PCoA plot of the weighted UniFrac distances between the piglets from both dietary treatments (Fig. 2).

### Taxonomic Classification of the Gut Microbiota of Lactating Sows and Their Piglets Using 16S rRNA Gene Sequencing

**Fig. 3:**
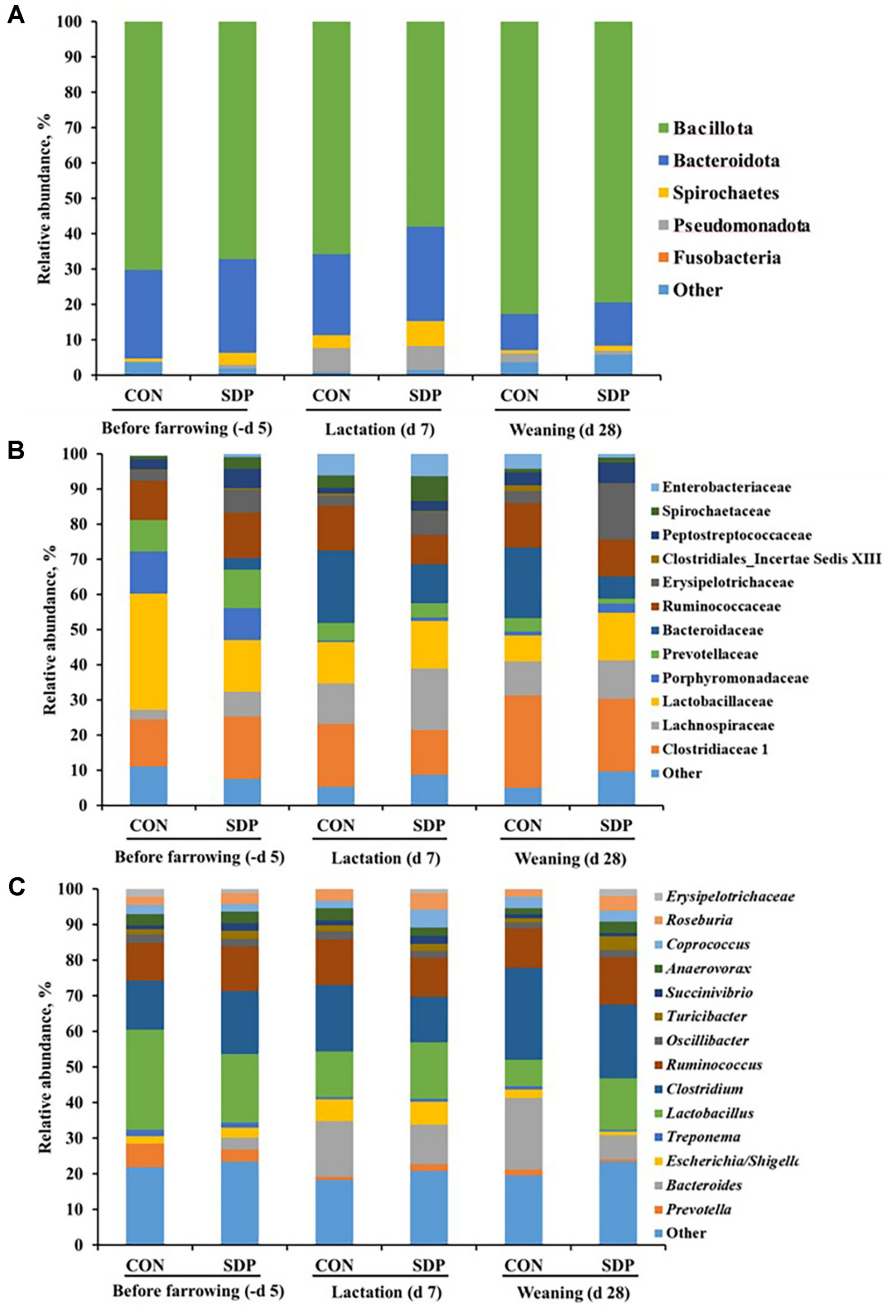
Taxonomic classification of the total bacteria based on the 16S rRNA gene sequences at the phylum (A), family (B), and genus (C) levels for the sow fecal samples. *n* = 3; CON, control diet; SDP, CON added with 1% dietary SDP.

As shown in Fig. 3, taxonomic classification of the fecal bacteria from the CON and SDP sow groups at the phylum, family, and genus levels was obtained by 16S rRNA gene sequencing. At the phylum level, the gut microbiota of the sow fecal samples mainly consisted of the phyla Bacillota and Bacteroidota and a higher relative abundance of phyla Bacteroidota was present in the sows fed the SDP diet than those fed the CON diet during the experimental period. Moreover, the composition of the phyla Spirochaetes (7.08% vs. 3.62%) in the SDP group was higher than that in the CON group at d 7 of lactation (Fig. 3A). At the family level, the result showed that the sows fed the SDP diet had an increased relative abundance of Lactobacillaceae (13.46% vs. 11.73% at d 7; 13.51%vs. 7.40% at d 28) and Erysipelotrichaceae (6.57% vs. 2.77% at d 7; 15.89% vs. 3.42% at d 28), and a decreased relative abundance of Clostridiaceae 1 (17.88% vs. 12.73% at d 7; 26.26% vs. 20.69% at d 28, *p* < 0.05) and Bacteroidaceae (20.66% vs. 11.09% at d 7; 20.15% vs. 6.30% at d 28) compared with those fed the CON diet (Fig. 3B). At the genus level, the sows fed the SDP diet had a higher relative abundance of *Lactobacillus* (14.51% vs. 7.40%, *p* < 0.05) and *Ruminococcus* (13.25% vs. 11.08%, *p* < 0.05) and a lower relative abundance of *Bacteriodes* (20.15% vs. 6.83%, *p* < 0.05), *Escherichia/Shigella* (2.28% vs. 1.07%, *p* < 0.05), and *Clostridium* (25.86% vs. 20.69%, *p* < 0.05) than those fed the CON diet at weaning (d 28) (Fig. 3C).

**Fig. 4:**
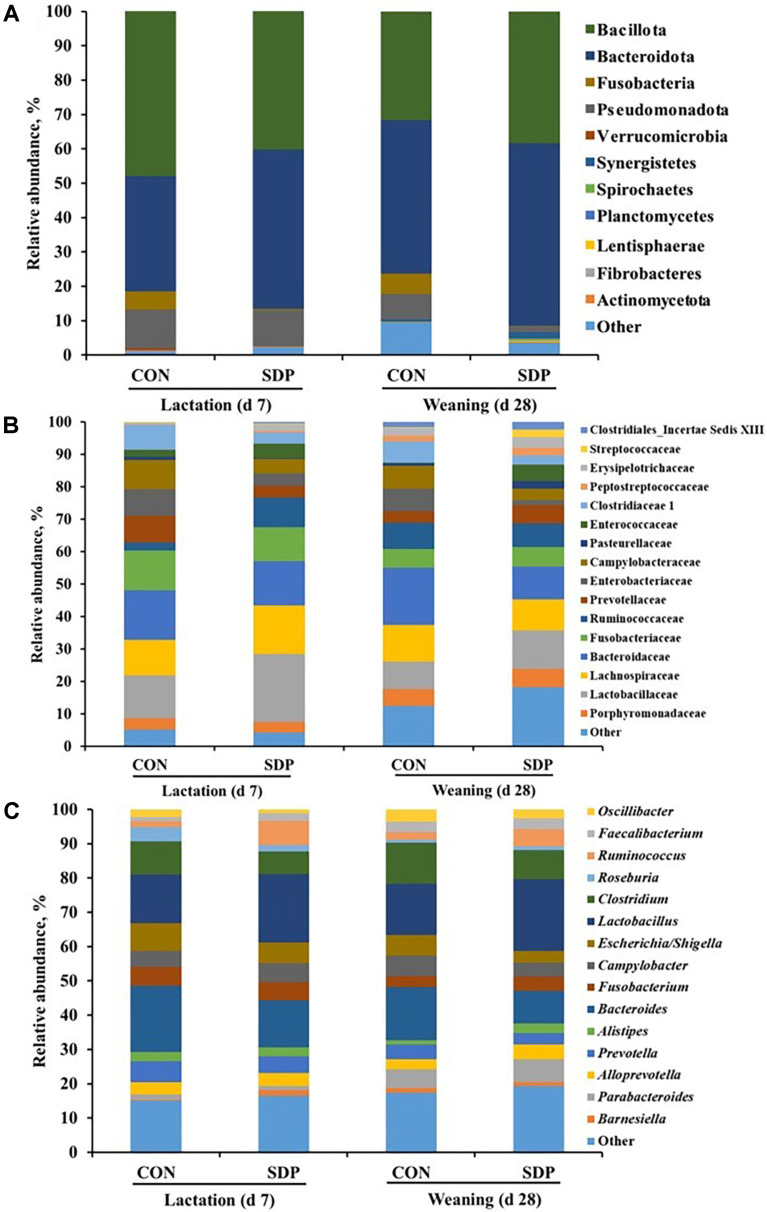
Taxonomic classification of the total bacteria based on the 16S rRNA gene sequences at the phylum (A), family (B), and genus (C) levels for the piglet fecal samples. *n* = 3; CON, control diet; SDP, CON added with 1% dietary SDP.

Similar profiles were observed in the sows and their piglets when comparing the taxa of fecal bacterial communities (Fig. 4). The relative abundance of phylum Bacteroidota in the piglets from the SDP group was higher than that of the piglets from the CON group during the experimental period at the phylum level (Fig. 4A). At the family level, the relative abundance decreased in Bacteroidaceae(15.35% vs. 13.69% at d 7; 17.55% vs. 10.03% at d 28), Enterobacteriaceae(8.18% vs. 3.72% at d 7; 6.97% vs. 1.44% at d 28), and Clostridiaceae 1 (7.60%vs. 3.43% at d 7; 6.63% vs. 2.88% at d 28, *p* < 0.05), while the relative abundance of Lactobacillaceae (20.95% vs. 13.20% at d 7; 11.89% vs. 8.48% at d 28, *p* < 0.05) increased in the piglets from the SDP group compared with that of the piglets from the CON group (Fig. 4B). Similar to the genus level results for the SDP sow group, relative abundance of *Lactobacillus* (19.95% vs. 14.20% at d 7; 20.89% vs. 15.01% at d 28, *p* < 0.05), and *Ruminococcus* (7.00% vs. 1.64% at d 7; 5.05% vs. 2.12% at d 28, *p* < 0.05) increased, whereas the relative abundance of *Bacteroides* (19.27% vs. 13.69% at d 7; 15.55% vs. 9.54% at d 28, *p* < 0.05), *Escherichia/Shigella* (8.08% vs. 6.01% at d 7; 5.94% vs. 3.44% at d 28, *p* < 0.05), and *Clostridium* (9.65% vs. 6.60% at d 7; 11.99% vs. 8.50% at d 28, *p* < 0.05) decreased in the piglets when the SDP diet was introduced (Fig. 4C).

## Discussion

Several studies have reported improvements in growth performance and intestinal health when SDP was used as a feed additive [14, 16, 24]. In a recent study, the effects of spray-dried porcine plasma as a prebiotic were confirmed by changes in the intestinal microbiota [25]. The addition of SDP increased the number of *Lactobacilli*, which was accompanied by the suppression of pathogenic bacteria and reduced the population of *E. coli* in the ileal and cecal digesta . We hypothesized that the addition of SDP to the diets of lactating sows would alter the gut microbial composition of the sows and their piglets. This is because the gastrointestinal tract of neonatal piglets is maternally independent, whereas the gut microbiota composition is influenced by the initial environment of the sow, such as the vagina, feces, and skin.

Our PCoA analysis of the fecal microbial communities of the sows showed a distinct separation between the CON and SDP groups during lactation. This indicates that the gut microbial composition of sows was affected by the dietary treatment with the addition of SDP. In our previous SDP study of sow productivity, we confirmed the results of improved productivity and immune response [13]. This evidence supports the results that feeding a SDP additive diet to sows is related to sow productivity by positively altering the composition of the gut microbiota. This study demonstrated that a high relative abundance of the phylum Bacteroidota was present in the fecal samples from sows and their piglets in the SDP group. Bacteroidota are recognized as bacteria with the ability to digest otherwise indigestible dietary compounds to provide additional energy to the host [26]. Moreover, several studies have reported that Bacteroidota produce short-chain fatty acids (SCFAs), which play an important role in gut health, provide energy to the intestinal mucosa, and promote the growth of epithelial cells [27, 28]. The relative abundance of the phylum Spirochaetes was also increased when the sows were fed the SDP diet during lactation. Spirochaetes have been reported to be related to the digestibility of dietary fiber because they break down hemicelluloses such as cellulose and xylan [29, 30]. The fecal microbial composition of the piglets in the SDP group showed a high relative abundance of the phylum Bacteroidota, similar to the results of the sows. These results suggest that the microbial composition of the sows can be transferred to their piglets and positively affect the digestibility of dietary fiber.

It was observed that the sow microbiota in the SDP group had a significantly higher relative abundance of the family Lactobacillaceae and genus *Lactobacillus*. They are bacteria well known for the production of lactic acid during carbohydrate fermentation [31-33]. Previous studies have confirmed that *Lactobacillus* species have specific probiotic traits which act against pathogenic microorganisms such as *E. coli* in the intestine [34-36] . Our results also showed that sows fed the SDP diet during lactation had an increased relative abundance of the family Erysipelotrichaceae and genus *Ruminococcaceae*. The enteric Erysipelotrichaceae family has been reported to be associated with host lipid metabolism in nutritional studies [37, 38]. Generally, *Ruminococcaceae* are recognized as butyrate- and other SCFA-producing bacteria with functional capacities related to maintaining intestinal health [39, 40]. Our results suggest that the pathogenic microbial communities such as the genus *Bacteroides*, *Escherichia/Shigella*, and *Clostridium* were decreased with the increase in these positive microbial communities.

Although a previous study reported the presence of bacteria in the placenta and amniotic fluid [41], it is unclear whether the early development of the gut microbiome of piglets is influenced by the intrauterine microbiome during pregnancy or postnatal environment. Based on previous studies, two hypotheses for the formation of the initial gut microbial colonization of piglets were confirmed. First, it was shown that the gut microbiota of the sow is deeply involved in the formation of the piglet's gut microbiota and consequently affects the immune system development, growth performance, and survival of the piglets [42-44]. Another hypothesis is that the caring environment plays a crucial role in the function and composition of the gut microbiota during both the suckling and weaning periods [42, 45]. Our previous studies confirmed that the nutritional changes in sow diets affected the sow and piglet health and gut microbiota [46, 47]. The present study observed similar gut microbial profiles, with increases in the genus *Lactobacillus* and *Ruminococcus* and decreases in the genus *Bacteroides*, *Escherichia/Shigella*, and *Clostridium*, in both the sows and piglets when the sows were fed a SDP additive diet during lactation. Therefore, our results suggest that the nutritional changes during lactation, such as SDP intake in sows, affect the development of the gut microbiome of piglets.

## Conclusion

In conclusion, we observed that the gut microbiota composition of lactating sows was modified by the addition of 1% SDP to the sow diet from late gestation until weaning. In addition, it was confirmed that the changes in the composition of the gut microbiota of the piglets changed in a similar pattern to that of the sows. As such, this data suggests that the addition of SDP to the sow diet during lactation altered the gut environment with positive microbial composition changes. We believe that these results provide improved nutritional control of sow diets during lactation which may contribute to the intestinal health and growth of piglets after weaning.

## Figures and Tables

**Table 1:** Composition of experimental diets of sows during late gestation and lactation.^1^

Items	Gestation diet		Lactation diet	
	CON	SDP	CON	SDP
Ingredient, %				
Corn	75.82	76.72	65.54	66.53
Soybean meal, 45%	21.30	19.40	31.81	29.82
SDP	-	1.00	-	1.00
Limestone	0.90	0.90	0.85	0.85
MDCP	1.58	1.58	1.40	1.40
Vitamin premix^2^	0.20	0.20	0.20	0.20
Mineral premix^3^	0.20	0.20	0.20	0.20
Total	100	100	100	100
Calculated composition				
Metabolizable energy, Mcal/kg	3.32	3.32	3.43	3.43
Crude protein, %	15.86	15.82	19.76	19.72
Crude fat, %	3.09	3.05	2.86	2.80
Crude fiber, %	2.97	3.00	3.33	3.36
NDF, %	8.71	8.78	10.78	10.81
ADF,%	4.18	4.20	4.63	4.65
Calcium, %	0.77	0.77	0.75	0.75
Phosphorus, %	0.64	0.65	0.65	0.65
Lysine, %	0.74	0.76	1.02	1.02
Methionine, %	0.25	0.25	0.30	0.30
Threonine, %	0.58	0.60	0.74	0.76
Tryptophan, %	0.16	0.16	0.22	0.22

^1^Sows were fed the dietary treatments from d 84 of gestation until farrowing and lactation before weaning.

^2^Provided per kg of diet: vitamin A, 10,000 IU; vitamin D_3_, 2,000 IU; vitamin E, 48 IU; vitamin K_3_, 1.5 mg; riboflavin, 6 mg; niacin, 40 mg; D-pantothenic acid, 17 mg; biotin, 0.2 mg; folic acid, 2 mg; choline, 166 mg; vitamin B_6_, 2 mg; and vitamin B_12_, 28 μg.

^3^Provided per kg of diet: Fe, 90 mg from iron sulfate; Cu, 15 mg from copper sulfate; Zn, 50 mg from zinc oxide; Mn, 54 mg from manganese oxide; I, 0.99 mg from potassium iodide; Se, 0.25 mg from sodium selenite.

ADF, acid detergent fiber; CON, control diet; MDCP, monodicalcium phosphate; NDF, neutral detergent fiber; SDP, dietary spray-dried plasma

**Table 2:** Alpha diversity analysis of the sow fecal microbiota using 16S rRNA gene sequencing.^1^

Period	Treatments	Sequence reads	OTUs	Chao1	Shannon	Simpson
Before farrowing (-d 5)	CON	3,382.67 ± 1,031.79	202.37 ± 19.30	254.77 ± 4.74	4.84 ± 1.83	0.80 ± 0.26
	SDP	4,093.00 ± 2,286.04	215.67 ± 27.43	259.87 ± 5.70	5.45 ± 0.31	0.94 ± 0.01
	*P*-value	0.649	0.539	0.299	0.598	0.429
Lactation (d 7)	CON	3,280.00 ± 1,553.52	134.67 ± 36.09	171.14 ± 46.18	4.13 ± 0.75	0.83 ± 0.06
	SDP	1,688.33 ± 204.84	125.33 ± 34.59	169.99 ±40.09	4.65 ± 1.14	0.89 ± 0.06
	*P*-value	0.153	0.763	0.976	0.543	0.288
Weaning (d 28)	CON	1,585.00 ± 108.97	110.00 ± 22.65	133.44 ± 26.94	4.34 ± 0.80	0.84 ± 0.10
	SDP	2,016.00 ± 419.40	139.67 ± 22.94	189.89 ± 35.89	4.82 ± 0.52	0.91 ± 0.05
	*P*-value	0.160	0.186	0.095	0.434	0.335

^1^Each value is the mean of 3 replicates per treatment.

CON, control diet; SDP, CON added with 1% dietary SDP; SEM, standard error of the mean; OTUs, operational taxonomic units.

**Table 3:** Alpha diversity analysis of the piglet fecal microbiota using 16S rRNA gene sequencing.^1^

Period	Treatments	Sequence reads	OTUs	Chao 1	Shannon	Simpson
Lactation (d 7)	CON	2,613.67 ± 838.39	66.67 ± 18.15	76.63 ± 19.90	3.69 ± 0.69	0.84 ± 0.08
	SDP	1,775.83 ± 319.59	58.83 ± 27.63	72.04 ± 39.11	3.74 ± 0.87	0.84 ± 0.09
	*P*-value	0.045	0.575	0.802	0.793	0.219
Weaning (d 28)	CON	1,966.00 ± 465.57	100.50 ± 33.30	117.55 ± 35.25	4.52 ± 0.91	0.89 ± 0.07
	SDP	2,324.67 ± 1,926.80	102.17 ± 42.89	119.43 ± 49.07	4.30 ± 0.74	0.85 ± 0.09
	*P*-value	0.634	0.942	0.941	0.655	0.481

^1^Each value is the mean of 6 replicates per treatment.

CON, control diet; SDP, CON added with 1% dietary SDP; SEM, standard error of mean; OTUs, operational taxonomic units.
